# Mixed Features in Recurrent Major Depressive Disorder: Prevalence, Risk Factors, and Clinical Consequences

**DOI:** 10.1155/da/7452200

**Published:** 2026-08-30

**Authors:** C. Hyung Keun Park, Chanhee Park, Sang Jin Rhee, Sooyeon Min, Yoojin Song, Heon-Jeong Lee, Seunghee Won, Kyu Young Lee, Dongyun Lee, Do Hoon Kim, Ji Hyun Baek, Young-Hoon Ko, Kwang-Yeon Choi, Young Tak Jo, Sung Joon Cho, Won Sub Kang, Young Min Choe, Sungwon Roh, Chun Il Park, Seoyoung Yoon, Hyung-Jun Yoon, Joonho Choi, Sangho Shin, Su Jeong Seong, Hyeon Ah Lee, Jonathan Flint, Yong Min Ahn, Kenneth S. Kendler

**Affiliations:** ^1^ Department of Psychiatry, Asan Medical Center, 88, Olympic-ro 43-gil, Songpa-gu, Seoul 05505, Republic of Korea, amc.seoul.kr; ^2^ Asan Institute for Life Sciences, Asan Medical Center, 88, Olympic-ro 43-gil, Songpa-gu, Seoul, Republic of Korea, amc.seoul.kr; ^3^ Department of Neuropsychiatry, Seoul National University Hospital, 101, Daehak-ro, Jongno-gu, Seoul, Republic of Korea, snuh.org; ^4^ Department of Psychiatry, Seoul National University College of Medicine, 103, Daehak-ro, Jongno-gu, Seoul, Republic of Korea, snuh.org; ^5^ Department of Psychiatry, Kangwon National University Hospital, 156, Baengnyeong-ro, Chuncheon-si, Gangwon-do, Republic of Korea, knuh.or.kr; ^6^ Department of Psychiatry, Korea University College of Medicine, 73, Goryeodae-ro, Seongbuk-gu, Seoul, Republic of Korea, korea.ac.kr; ^7^ Department of Psychiatry, School of Medicine, Kyungpook National University, 680, Gukchaebosang-ro, Jung-gu, Daegu, Republic of Korea, knu.ac.kr; ^8^ Department of Psychiatry, Nowon Eulji University Hospital, 68, Hangeulbiseok-ro, Nowon-gu, Seoul, Republic of Korea; ^9^ Department of Neuropsychiatry, School of Medicine, Eulji University, 77, Gyeryong-ro 771beon-gil, Jung-gu, Daejeon, Republic of Korea, eulji.ac.kr; ^10^ Department of Psychiatry, Gyeongsang National University Changwon Hospital, Gyeongsang National University School of Medicine, 816, Jinju-daero, Jinju-si, Gyeongsangnam-do, Republic of Korea, gnu.ac.kr; ^11^ Department of Psychiatry, Chuncheon Sacred Heart Hospital and Mind-Neuromodulation Laboratory, Hallym University College of Medicine, 77, Sakju-ro, Chuncheon-si, Gangwon-do, Republic of Korea, hallym.ac.kr; ^12^ Department of Psychiatry, Samsung Medical Center, Sungkyunkwan University School of Medicine, 81, Irwon-ro, Gangnam-gu, Seoul, Republic of Korea, skkumed.ac.kr; ^13^ Department of Psychiatry, Korea University College of Medicine, Korea University Ansan Hospital, 123, Jeokgeum-ro, Danwon-gu, Ansan-si, Gyeonggi-do, Republic of Korea, kumc.or.kr; ^14^ Department of Psychiatry, College of Medicine, Chungnam National University, 282, Munhwa-ro, Jung-gu, Daejeon, Republic of Korea, cnu.ac.kr; ^15^ Department of Psychiatry, Kangdong Sacred Heart Hospital, Hallym University College of Medicine, 150, Seongan-ro, Gangdong-gu, Seoul, Republic of Korea, hallym.ac.kr; ^16^ Department of Psychiatry, Kangbuk Samsung Hospital, Sungkyunkwan University School of Medicine, 29, Saemunan-ro, Jongno-gu, Seoul, Republic of Korea, skkumed.ac.kr; ^17^ Workplace Mental Health Institute, Kangbuk Samsung Hospital, 29, Saemunan-ro, Jongno-gu, Seoul, Republic of Korea; ^18^ Department of Psychiatry, College of Medicine, Kyung Hee University, 26, Kyungheedae-ro, Dongdaemun-gu, Seoul, Republic of Korea, khu.ac.kr; ^19^ Department of Neuropsychiatry, Hallym University Dongtan Sacred Heart Hospital, 7, Keunjaebong-gil, Hwaseong-si, Gyeonggi-do, Republic of Korea, hallym.or.kr; ^20^ Department of Psychiatry, Hanyang University College of Medicine, 222, Wangsimni-ro, Seongdong-gu, Seoul, Republic of Korea, hanyang.ac.kr; ^21^ Department of Psychiatry, Yonsei University College of Medicine, 50-1, Yonsei-ro, Seodaemun-gu, Seoul, Republic of Korea, yonsei.ac.kr; ^22^ Institute of Behavioral Science in Medicine, Yonsei University College of Medicine, 50-1, Yonsei-ro, Seodaemun-gu, Seoul, Republic of Korea, yonsei.ac.kr; ^23^ Department of Psychiatry, Daegu Catholic University School of Medicine, 33, Duryugongwon-ro 17-gil, Nam-gu, Daegu, Republic of Korea, cu.ac.kr; ^24^ Department of Psychiatry, College of Medicine, Chosun University, 309, Pilmun-daero, Dong-gu, Gwangju, Republic of Korea, chosun.ac.kr; ^25^ Department of Psychiatry, Hanyang University Guri Hospital, 153, Gyeongchun-ro, Guri-si, Gyeonggi-do, Republic of Korea, hanyang.ac.kr; ^26^ Department of Psychiatry, Konyang University Hospital, 158, Gwanjeodong-ro, Seo-gu, Daejeon, Republic of Korea, konyang.ac.kr; ^27^ Department of Psychiatry, College of Medicine, Konyang University, 158, Gwanjeodong-ro, Seo-gu, Daejeon, Republic of Korea, konyang.ac.kr; ^28^ Department of Psychiatry, Soonchunhyang University Cheonan Hospital, Soonchunhyang University College of Medicine, 31, Suncheonhyang 6-gil, Dongnam-gu, Cheonan-si, Chungcheongnam-do, Republic of Korea, sch.ac.kr; ^29^ Department of Psychiatry and Biobehavioral Sciences, University of California, 760 Westwood Plaza, Los Angeles, California, 90024, USA, berkeley.edu; ^30^ Department of Psychiatry, Virginia Institute for Psychiatric and Behavioral Genetics, Virginia Commonwealth University, 800 E. Leigh Street, Richmond, Virginia, 23219, USA, vcu.edu

**Keywords:** KOMOGEN-D, Korea, major depressive disorder, mixed features, women

## Abstract

**Objectives:**

Mixed features in major depressive disorder (MDD)—the presence of manic symptoms during major depressive episodes (MDEs)—are associated with adverse clinical outcomes. We investigated the prevalence and clinical correlates of mixed features among Korean women with recurrent MDD.

**Method:**

Data (*n* = 3000) were drawn from the Korean Mood Disorder Genetic Study‐Depression, a nationwide case‐control study recruiting women aged ≥30 years with recurrent MDD from 47 hospitals across South Korea. Structured clinical interviews assessing depressive symptoms, family history, comorbidities, and psychosocial functioning were administered. Mixed features during MDEs were defined per Diagnostic and Statistical Manual of Mental Disorders (DSM)‐5 criteria. Generalized linear models (GLMs) examined associations between mixed features and clinical correlates.

**Results:**

The worst‐MDE‐based prevalence of mixed features was 4.6%. A family history of manic episodes (odds ratio [OR] = 3.36, 95% confidence interval [CI]: 1.67–6.36) and of MDEs (OR = 1.90, 95% CI: 1.41–2.55) was significantly associated with mixed features. Five worst‐MDE symptoms were independently associated with mixed features: increased weight (OR = 2.19, 95% CI: 1.16–4.09), late insomnia (OR = 1.84, 95% CI: 1.07–3.41), slow or mixed thoughts (OR = 1.93, 95% CI: 1.18–3.33), suicidal ideation (OR = 1.96, 95% CI: 1.04–3.94), and diurnal mood variation (OR = 1.94, 95% CI: 1.34–2.81). Mixed features were associated with mood‐stabilizer use (lifetime use OR = 2.21, 95% CI: 1.38–3.42; current use OR = 2.53, 95% CI: 1.58–3.92), panic disorder (OR = 2.10, 95% CI: 1.46–2.99), premenstrual syndrome symptoms (rate ratio [RR] = 1.22, 95% CI: 1.08–1.37), and damage by suicide attempts (RR = 1.66, 95% CI: 1.17–2.07).

**Conclusions:**

Although less prevalent in this clinically ascertained sample than in Western samples, mixed features in recurrent MDD exhibit distinct clinical patterns. This finding underscores the importance of screening for mixed features, particularly when associated depressive symptoms are present, to inform early detection and treatment planning.

## 1. Introduction

Kraepelin was the first to conceptualize a detailed concept of mixed states, describing combinations of manic and depressive symptoms—such as depressive mania and excited depression—within his framework of manic–depressive insanity [1]. Despite this early work, mixed states remained under‐recognized until the publication of the Diagnostic and Statistical Manual of Mental Disorders (DSM)‐III [2]. DSM‐III defined mixed episodes as the simultaneous presence of syndromal manic and major depressive episodes (MDEs) and classified them solely as subtypes of bipolar disorder [3]. This diagnostic structure persisted through DSM‐IV [4].

However, the ecological validity of this framework has increasingly been questioned. In clinical practice, fully concurrent syndromal presentations of both mood episodes are uncommon [5]. To address these limitations, DSM‐5 removed the mixed episode diagnosis and introduced the “with mixed features” specifier [6]. This revision produced two major changes: (1) the specifier was expanded to include major depressive disorder (MDD), recognizing that mixed presentations occur beyond bipolar disorder and (2) full criteria for both mood episodes were no longer required. Instead, the mixed features specifier for MDEs requires at least three nonoverlapping symptoms, such as expansive or elevated mood, grandiosity, talkativeness, racing thoughts/flight of idea, increased energy or goal‐directed activity, increased involvement in activities having a high potential for painful consequences, or decreased need for sleep [7].

Western studies indicate that a substantial proportion of individuals with MDD experience mixed features. Among 2397 outpatients with non‐psychotic MDD, 9.6% met DSM‐5 criteria for mixed features [8]. Another study in the United States similarly showed a prevalence of 15.5% [9]. These presentations have important clinical implications: mixed features are associated with longer MDE duration, suicidal ideation, suicide attempts, and impaired social functioning, even after adjusting for depression severity [9]. Likewise, a large‐scale study of over 100,000 patients with MDD reported higher rates of comorbid anxiety and substance abuse, increased likelihood of hospitalization, and greater healthcare costs among patients with mixed features [10]. Mixed features may also serve as a risk factor for progression to bipolar disorder [11, 12]. In a longitudinal cohort, hypomanic symptoms in unipolar depression were significantly associated with subsequent conversion to bipolar disorder [13].

Given the association of mixed features with adverse outcomes and bipolar conversion risk [9, 10, 13], early detection and tailored interventions are essential [5]. Although clinicians are encouraged to assess hypomanic symptoms thoroughly in patients experiencing MDEs [5], time constraints in routine clinical practice limit the feasibility of such assessments, contributing to the underdiagnosis of mixed features. Identifying risk factors that may facilitate early detection is essential.

Research in bipolar disorder has highlighted specific symptom correlates—such as insomnia, poor concentration, decreased libido, and poor appetite—that may accompany MDE with psychomotor agitation [14]. However, relatively few studies have examined which clinical symptoms are associated with DSM‐5–defined mixed features in patients with MDD.

Furthermore, a recent meta‐analysis revealed marked cross‐cultural variations in the prevalence of mixed features [7]. While Western studies report prevalence rates of 9.6%–15.5%, East Asian studies show substantially lower rates, with 2.2% (12/552) in a Korean clinical sample [15]. These disparities highlight the need for large‐scale investigations of mixed features in East Asian populations with MDD as well as examination of associations between mixed features and various mood‐related symptoms. As relationships between mixed features and lifetime illness course, psychotropic use patterns, and psychosocial functioning have been studied primarily in Western populations, cross‐cultural validation in East Asian contexts is warranted.

Given these substantial cross‐cultural differences and the limited evidence from East Asian populations, the present study examined mixed features among Korean women with recurrent MDD. As prior research has shown sex‐specific genetic effects in MDD [16], the study focused exclusively on women with four Korean grandparents to reduce genetic heterogeneity and enhance phenotypic precision. Specifically, we aimed (1) to determine the prevalence of mixed features among 3000 patients with recurrent MDD recruited from multiple centers across South Korea; (2) to examine associations between specific mood‐related symptoms during the worst MDE and the presence of mixed features; and (3) to explore relationships between mixed features and lifetime illness course, psychotropic use, and psychosocial functioning.

## 2. Methods

### 2.1. Study Participants

Data for this study were obtained from the Korean Mood Disorder Genetic Study–Depression (KOMOGEN‐D), an ongoing genome‐wide association study of MDD. The current analysis included 3000 participants recruited from 47 hospitals across South Korea. Eligibility criteria included women with four Korean grandparents, aged 30 years or older, with a history of at least two MDEs and the first episode occurring between ages 14 and 50. Exclusion criteria were any history of bipolar disorders, psychotic disorders, intellectual disability, mild cognitive impairment, dementia, current prescription of cognitive enhancers, and current or past alcohol or substance use disorder.

Participants completed a comprehensive 1.5 h assessment. Interviews were conducted by trained registered nurses, clinical psychologists, psychiatrists, or psychiatry residents who had completed at least 1 week of training provided by the KOMOGEN‐D team. The interview assessed psychopathology, demographic characteristics, personal history, and psychosocial functioning. All interviews were audio‐recorded with participants’ consent, and a subset was reviewed by trained editors for quality control.

Data were collected using the research electronic data capture (REDCap) system, a secure web‐based platform developed for research data collection and hosted at Seoul National University Hospital [17, 18]. Although interviews were primarily conducted in person, remote assessments (online or telephone) were permitted when in‐person evaluations were not possible due to legitimate circumstances.

The study protocol was approved by the Institutional Review Board at the University of California, Los Angeles; the ethics committees of all participating hospitals in Korea; and the Institutional Review Board of Asan Medical Center (2021‐1123).

### 2.2. Measures

Lifetime diagnoses of depressive disorders (MDD and dysthymia) were established using the Korean version of the Composite International Diagnostic Interview (CIDI 3.0) [19]. The worst MDE was identified based on participants’ self‐report; during the interview, participants were asked to identify the MDE they considered to be the most severe episode in their lifetime.

Mixed features during the worst MDE were assessed according to the DSM‐5 mixed‐features specifier. The depressive symptoms defining the worst MDE (e.g., sadness, emptiness, or depressed mood, and diminished interest in daily activities) were confirmed during the diagnostic interview not to be attributable to a general medical condition or to medication. For each participant, seven hypomanic symptoms were evaluated: (1) elevated or expansive mood; (2) inflated self‐esteem or grandiosity; (3) being more talkative than usual or pressure to keep talking; (4) flight of ideas or the subjective experience that thoughts are racing; (5) increase in energy or goal‐directed activity (socially, at work or school, or sexually); (6) increased or excessive involvement in activities that have a high potential for painful consequences; and (7) decreased need for sleep. Mixed features were considered present when at least three of these symptoms co‐occurred during the worst MDE. To ensure that these symptoms were present concurrently with the worst MDE, each symptom was queried with reference to that specific period (i.e., “during that time, when things were at their worst”). Symptoms judged to be substance‐ or medication‐induced were excluded on the basis of the diagnostic interview.

Mixed features and their relationships with mood‐related symptoms during the worst MDE were assessed, including Beck’s cognitive triad (helplessness, hopelessness, and worthlessness), melancholic features, irritability, and anxiety. Suicidal ideation, suicide plan and attempts during the worst MDE were also evaluated. The assessment instrument was translated into Korean by bilingual psychiatrists and psychologists at Seoul National University Hospital, Seoul National University Bundang Hospital, and Samsung Medical Center and revised by the KOMOGEN‐D team. The precise wording of the diagnostic interview used to assess the hypomanic symptoms and various depressive symptoms is provided in Tables S1 and S2, respectively.

Associations between mixed features and clinical characteristics were also examined. First, lifetime history of psychiatric comorbidities was assessed using interview material from the Virginia Adult Twin Study of Psychiatric and Substance Use Disorders (VATSPSUD) [20], which evaluates seven anxiety disorders: generalized anxiety disorder, panic disorder, and five phobia subtypes (agoraphobia, social phobia, situational phobia, animal phobia, and blood‐injury phobia). Specific phobia was defined as the presence of any phobia subtypes. Symptoms of premenstrual syndrome were also assessed using a brief assessment of premenstrual syndrome [21].

Second, psychotropic use was assessed for both lifetime and current use, including antipsychotics for psychotic symptoms, mood stabilizers, antidepressants, and other psychotropics. Psychiatric hospitalization history and clinical features of MDD (e.g., number of lifetime MDEs and duration of the longest MDE) were assessed through comprehensive clinical assessment. Suicidality measures included the number of suicide attempts and the degree of damage from the most serious attempt. The current mood state was evaluated using the depression items of the Symptom Checklist‐90 [22]. Neuroticism was measured using the 23‐item Eysenck Personality Questionnaire [23].

Third, social support was evaluated using a social interaction scale [24], which measures positive and negative interactions with friends (2 and 3 items), relatives (2 and 3 items), and a spouse (4 items each for positive and negative interactions). Cronbach’s *α* values were 0.65 and 0.70 (friends), 0.80 and 0.70 (relatives), and 0.90 and 0.82 (spouses). Additional social functioning indicators included the frequency of contact with friends and relatives, number of friends, and frequency of participation in clubs or social groups.

### 2.3. Statistical Analysis

Analyses were performed using R 4.4.1 [25]. The proportion of the sample with mixed features during the worst MDE was calculated. Mixed features were coded as a binary variable (0 = absent and 1 = present). To identify depressive symptoms associated with mixed features, we tested the association between mixed features and every mood‐related symptom assessed during the worst MDE that was captured by our questionnaire, excluding psychotic symptoms. Each symptom was first examined in a univariate model, and all symptoms showing a significant association were subsequently entered into the multivariate logistic regression model. To examine associations between individual mood‐related symptoms during the worst MDE and the presence of mixed features, we conducted univariate logistic regression analyses. To control for multiple comparisons and reduce Type I error, false discovery rate (FDR) correction was applied using the Benjamini–Hochberg method; statistical significance was defined as *q*‐values <0.05.

All symptoms showing significant associations in the univariate analyses were entered into a multivariable logistic regression model using the enter method, with the presence or absence of mixed features as the outcome variable. To examine associations between mixed features and lifetime clinical characteristics, generalized linear models (GLMs) were applied. Specifically, we tested whether the presence of mixed features was significantly associated with clinical characteristics, including lifetime and current use of psychotropics, psychiatric comorbidity, suicidality, current mood state, and psychosocial functioning. Depending on the distribution of each dependent variable, GLMs with appropriate link functions were selected, and age was included as a covariate. For count data, quasi‐Poisson regression was used instead of standard Poisson link functions because the quasi‐Poisson model allows the dispersion parameter to vary rather than fixing it at 1, thereby accounting for overdispersion in the data and reducing false‐positive rates.

## 3. Results

The worst‐MDE‐based prevalence of mixed features among Korean women with recurrent MDD was 4.6% (*n* = 136). A family history of manic episodes significantly predicted mixed features during the worst MDE (odds ratio [OR] = 3.36, 95% confidence intervals [CIs] = 1.67–6.36, *q* < 0.001) and a greater number of mixed features symptoms (rate ratio [RR] = 1.79, 95% CI = 1.24–2.53, *q* = 0.02). Similarly, the presence of family members with a history of MDEs significantly predicted both mixed features during the worst MDE (OR = 1.90, 95% CI = 1.41–2.55, *q* < 0.001) and a greater number of mixed features symptoms (RR = 1.30, 95% CI = 1.13–1.50, *q* < 0.001) (Tables 1 and 2).

**Table 1:** Family history of mood episodes and the mixed features (binary).

Family history of mood episodes	OR [95% CI]	*p*	*q*
Family history of manic episode	3.36 [1.67, 6.36]	<0.001	<0.001
Family history of major depressive episode	1.90 [1.41, 2.55]	<0.001	<0.001

*Note:* Age was included as a covariate. The number of informatives was weighted. The false discovery rate (*q*‐value) is reported for each *p*‐value. A significance threshold of *q* < 0.05 was used.

Abbreviations: CI, confidence interval; OR, odds ratio.

**Table 2:** Family history of mood episodes and the mixed features (count).

Family history of mood episodes	RR [95% CI]	*p*	*q*
Family history of manic episode	1.79 [1.24, 2.53]	0.02	0.02
Family history of major depressive episode	1.30 [1.13, 1.50]	<0.001	<0.001

*Note:* Age was included as a covariate. The number of informatives was weighted. The false discovery rate (*q*‐value) is reported for each *p*‐value. A significance threshold of *q* < 0.05 was used.

Abbreviations: CI, confidence interval; RR, rate ratio.

**Figure 1:**
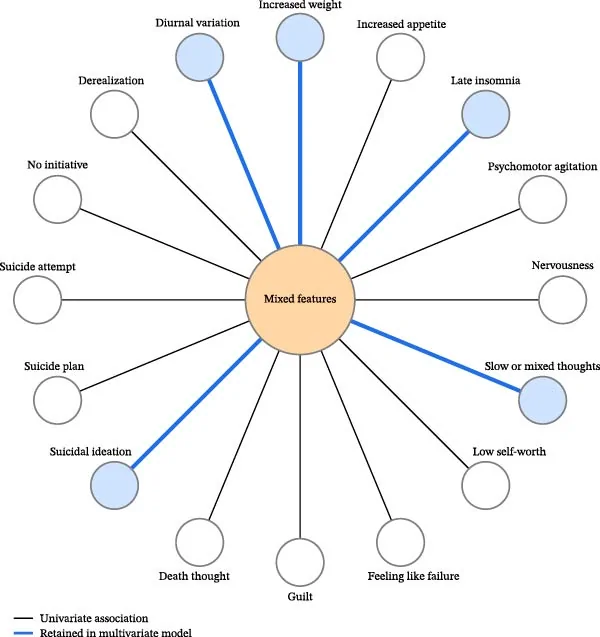
Network plot of mood‐related symptoms associated with mixed features during the worst major depressive episode.

The mood‐related symptoms significantly associated with the presence of mixed features in the univariate GLM after FDR correction (*q* < 0.05) are presented in Table 3. The results for all mood‐related symptoms examined in the univariate GLM are presented in Table S3. These symptoms were subsequently entered into multivariable logistic regression analysis. Five symptoms emerged as significant predictors of mixed features during the worst MDE: increased weight (OR = 2.19, 95% CI = 1.16–4.09, *p* = 0.02), late insomnia (OR = 1.84, 95% CI = 1.07–3.41, *p* = 0.04), slow or mixed thoughts (OR = 1.93, 95% CI = 1.18–3.33, *p* = 0.01), suicidal ideation (OR = 1.96, 95% CI = 1.04–3.94, *p* < 0.05), and diurnal mood variation (worse mood in the morning) (OR = 1.94, 95% CI = 1.34–2.81, *p* < 0.001) (Table 4 and Figure 1). Model fit was acceptable, and collinearity diagnostics indicated no multicollinearity problem in the model (AIC = 965.00, Nagelkerke pseudo‐*R* ^2^ = 0.18, and all VIF ≤2.15; Table S4).

**Table 3:** Univariate logistic regression testing the association between the mood‐related symptoms and the presence of mixed features in the worst major depressive episode.

Mood‐related symptoms in the worst MDE	Mixed features		Univariate logistic regression		
	Present (*N* = 136)	Absent (*N* = 2796)	OR [95% CI]	*p*	*q*
Increased weight	47	433	2.92 [2.00, 4.20]	<0.001	<0.001
Increased appetite	45	474	2.41 [1.65, 3.47]	<0.001	<0.001
Late insomnia	121	2171	2.27 [1.36, 4.07]	<0.001	0.01
Psychomotor agitation	45	642	1.68 [1.15, 2.42]	<0.001	0.02
Nervousness	133	2543	4.32 [1.62, 17.62]	0.01	0.03
Slow or mixed thoughts	115	1918	2.70 [1.69, 4.54]	<0.001	<0.001
Low self‐worth	105	1836	1.75 [1.18, 2.68]	0.01	0.02
Feeling like failure	109	1971	1.74 [1.14, 2.74]	0.02	0.03
Guilt	122	2273	1.98 [1.17, 3.63]	0.02	0.04
Death thought	126	2321	2.57 [1.41, 5.26]	0.006	0.02
Suicidal ideation	111	1813	2.50 [1.63, 4.00]	<0.001	0.001
Suicide plan	77	1122	1.94 [1.37, 2.76]	<0.001	0.001
Suicide attempt	54	733	1.85 [1.30, 2.63]	0.001	0.003
Derealization	97	1509	2.09 [1.44, 3.09]	<0.001	0.001
Diurnal variation	66	1006	1.65 [1.17, 2.33]	0.003	0.01

*Note:* The false discovery rate (*q*‐value) is reported for each *p*‐value. A significance threshold of *q* < 0.05 was used. Totals may vary due to missing values in each variable. Only significant variables are retained in the table.

Abbreviations: CI, confidence interval; MDE, major depressive episode; OR, odds ratio.

**Table 4:** Multivariate logistic regression testing the association between the mood‐related symptoms and the presence of mixed features in the worst major depressive episode.

Variable	OR	95% CI		*p*
		LLCI	ULCI	
Increased weight	**2.19**	**1.16**	**4.09**	**0.02**
Increased appetite	1.05	0.55	2.00	0.88
Late insomnia	**1.84**	**1.07**	**3.41**	**0.04**
Agitation	1.32	0.88	1.96	0.17
Nervousness	2.66	0.97	10.98	0.10
Slow or mixed thoughts	**1.93**	**1.18**	**3.33**	**0.01**
Low self‐worth	1.05	0.65	1.71	0.85
Feeling like failure	0.93	0.57	1.58	0.79
Guilt	1.14	0.63	2.20	0.68
Death thought	0.90	0.40	2.13	0.80
Suicidal ideation	**1.96**	**1.04**	**3.94**	**0.05**
Suicide plan	1.18	0.76	1.85	0.46
Suicide attempt	1.23	0.81	1.87	0.33
Derealization	1.34	0.89	2.05	0.16
Diurnal variation	**1.94**	**1.34**	**2.81**	**<0.001**

*Note:* Age was included as a covariate. Significant results are in bold.

Abbreviations: CI, confidence interval; LL, lower limit, OR, odds ratio; UL, upper limit.

As shown in Table 5, among psychotropic use, the presence of mixed features was significantly associated with both lifetime and current use of mood stabilizers (OR = 2.21, 95% CI = 1.38–3.42, *q* = 0.001; OR = 2.53, 95% CI = 1.58–3.92, *q* < 0.001). Regarding psychiatric comorbidity, mixed features were significantly related to the lifetime histories of panic disorder (OR = 2.10, 95% CI = 1.46–2.99, *q* < 0.001) and phobia (OR = 2.45, 95% CI = 1.44–4.48, *q* = 0.004). The presence of mixed features was also associated with the number of premenstrual syndrome symptoms (RR = 1.22, 95% CI = 1.08–1.37, *q* = 0.001).

**Table 5:** Effects of generalized linear models testing for association between the presence of mixed features and lifetime clinical factors.

Variable	Estimate	95% CI		*p*	*q*
		LLCI	ULCI		
Lifetime use of psychotropics^a^					
Antipsychotics	0.82	0.20	2.24	0.79	0.88
Mood stabilizers	**2.21**	**1.38**	**3.42**	**<0.001**	**0.001**
Antidepressants	0.95	0.42	2.74	0.82	0.88
Other psychotropics	0.75	0.53	1.05	0.14	0.27
Current use of psychotropics					
Antipsychotics	0.82	0.20	2.24	0.80	0.88
Mood stabilizers	**2.53**	**1.58**	**3.92**	**<0.001**	**<0.001**
Antidepressants	1.14	0.61	2.35	0.86	0.89
Other psychotropics	0.80	0.56	1.13	0.29	0.43

Psychiatric comorbidity					
Dysthymia^a^	1.40	0.95	2.03	0.08	0.18
Premenstrual syndrome symptoms^b^	**1.22**	**1.08**	**1.37**	**<0.001**	**0.001**
Generalized anxiety disorder^a^	1.45	1.01	2.05	0.02	0.05
Panic^a^	**2.10**	**1.46**	**2.99**	**<0.001**	**<0.001**
Phobia^a^	**2.45**	**1.44**	**4.48**	**0.001**	**0.004**

Clinical factors					
Number of depressive episodes^b^	0.95	0.83	1.09	0.43	0.61
Duration of the longest depressive episode^b^	1.14	0.96	1.42	0.24	0.40
Current mood state^c^	5.57	2.39	8.40	0.46	0.63
Hospitalization^a^	1.27	0.97	1.14	0.22	0.40
Neuroticism^b^	**1.13**	**1.06**	**1.19**	**<0.001**	**<0.001**

Suicidality					
Number of suicide attempts^b^	1.13	0.96	1.32	0.90	0.90
Damage by suicide attempts^b^	**1.66**	**1.17**	**2.07**	**0.001**	**0.002**

Social life friends					
Frequency of seeing friends^c^	−0.20	−0.52	0.11	0.28	0.43
Number of friends^b^	0.99	0.77	1.02	0.11	0.18
Positive interaction^c^	0.05	−0.15	0.24	0.62	0.81
Negative interaction^c^	0.33	−0.03	0.62	0.07	0.16
Relatives					
Contact with relatives^c^	−0.02	−0.34	0.31	0.81	0.88
Positive interaction^c^	−0.21	−0.47	0.09	0.16	0.30
Negative interaction^c^	**0.62**	**0.20**	**1.05**	**0.001**	**0.005**
Spouse					
Positive interaction^c^	**−1.92**	**−3.17**	**−0.66**	**0.007**	**0.02**
Negative interaction^c^	**1.72**	**0.65**	**2.79**	**0.001**	**0.004**
Social club^c^	0.10	−0.21	0.42	0.66	0.83

*Note:* The false discovery rate (*q*‐value) is reported for each *p*‐value. A significance threshold of *q* < 0.05 was used. Significant results are in bold. Estimates were reported for the three types of regression models: linear regression (standardized parameter estimates, *β*); logistic regression (OR); and quasi‐Poisson regression (RR). ORs and RRs were calculated by exponentiating the regression coefficients.

Abbreviation: CI, confidence interval.

^a^Logistic regression was used.

^b^Quasi‐poisson regression was used.

^c^Linear regression was used.

In relation to suicidality, mixed features were significantly related to damage by suicide attempts (RR = 1.66, 95% CI = 1.17–2.07, *q* = 0.002). The presence of mixed features was positively associated with neuroticism (RR = 1.13, 95% CI = 1.06–1.19, *q* < 0.001). Regarding psychosocial functioning, mixed features were associated with more negative interactions with relatives (*β* = 0.62, *q* = 0.005), fewer positive interactions with spouse (*β* = −1.92, *q* = 0.02), and more frequent negative interactions with spouse (*β* = 1.72, *q* = 0.004).

To enhance the robustness of our analysis, we conducted sensitivity analyses examining the associations between the manic/hypomanic symptoms constituting mixed features and clinical characteristics, additionally controlling for current depression severity. The significant associations were fully retained (Table S5). Associations between the number of manic/hypomanic symptoms and clinical characteristics are presented in the Supporting Information (Table S6).

## 4. Discussion

This study yielded three main findings. First, 4.6% of Korean women with recurrent MDD met DSM‐5 criteria for mixed features during their worst MDE. Second, five symptoms within the worst MDE including increased weight, late insomnia, slow or mixed thought, suicidal ideation, and diurnal mood variation (worse mood in the morning) were uniquely associated with mixed features. Third, mixed features during the worst MDE were significantly associated with adverse clinical characteristics, including use of a mood stabilizer, lifetime anxiety disorders, premenstrual syndrome symptoms, suicidality, and interpersonal relationships.

The worst‐MDE‐based prevalence of mixed features in our cohort aligns with prior findings from East Asian populations, which have consistently reported lower rates compared to Western samples. Rates as low as 2.2% in Korea [15] and 0% in Japan [26] contrast sharply with the 9.6%–15.5% reported in Western studies [8, 9]. Cultural differences in emotional expression may also contribute, although we did not formally test this, to this discrepancy [27]. In East Asian cultures, emotional restraint has traditionally been considered a social norm [28], potentially reducing the likelihood that nonoverlapping manic symptoms—such as elevated mood, grandiosity, talkativeness, and decreased need for sleep—are expressed or reported during MDEs [7]. The DSM‐5 mixed feature criteria exclude commonly co‐occurring symptoms, such as irritability and distractibility, which may be more universally expressed across cultures [29, 30]. As a result, the criteria rely predominantly on symptoms that are more susceptible to cultural variation in expression and reporting, which may disproportionately attenuate the prevalence estimates in East Asian populations. These cultural and methodological factors highlight ongoing concerns regarding the cross‐cultural validity of DSM‐5 mixed feature criteria [31]. Additionally, recruiting only women with recurrent MDD may have yielded a more homogeneous depressive phenotype, further constraining the detection of mixed presentation.

Several mechanisms may underlie these East–West disparities, including cultural norms around emotional expression, cross‐cultural variation in the interpretation and application of diagnostic criteria, differences in the genetic background, and help‐seeking behavior [32, 33]. We emphasize, however, that we did not conduct a formal test of measurement equivalence, and our study cannot establish that the lower prevalence reflects cultural differences in symptom expression rather than other sources of variation. Prior cross‐cultural work is informative but not decisive: using multigroup confirmatory factor analysis of the HCL‐32 across 12 countries, Angst et al. [34] reported that manic‐symptom measurement was largely invariant across regions, yet a few items—including “jumping thoughts” and “higher mood”—functioned differently in the East Asian sample, indicating that identical symptom items may be expressed or interpreted differently across cultures. To our knowledge, no study has directly tested measurement invariance for the specific hypomanic symptoms comprising the DSM‐5 mixed‐features specifier across East Asian and Western populations. The observed prevalence gap may therefore also reflect differences in diagnostic criteria, sample composition, and recruitment strategy rather than true phenomenological differences alone. Future studies employing multigroup confirmatory factor analysis are needed to formally test the cross‐cultural measurement invariance of the mixed‐features criteria and to disentangle genuine differences in symptom expression from measurement artifacts.

Our finding that a family history of both manic and depressive episodes was associated with mixed features during the worst MDE is consistent with emerging evidence suggesting genetic bipolar traits in patients with MDD with mixed features. Prior work has shown elevated rates of bipolar family history among MDD patients with mixed features compared with those without [35]. This pattern suggests that mixed features in MDD may represent a phenotypic expression of an underlying bipolar genetic vulnerability.

The specific depressive symptoms identified in our study that were uniquely associated with mixed features may provide insights into the phenomenology of this clinical presentation. The association with increased weight, an atypical symptom, aligns with the evidence linking atypical features to bipolar depression and mixed features [36]. Consistent with a broader link between weight change and outcome in bipolar spectrum illness, clinically significant weight gain following a first manic episode has been shown to predict poorer global functioning independently of concurrent mood symptoms, suggesting that weight‐related features may carry prognostic and potentially modifiable clinical significance [37]. Similarly, the associations with late insomnia and diurnal mood variation (worse mood in the morning)—both melancholic features—are noteworthy, as melancholic symptoms are frequently observed in bipolar depression [38]. These melancholic and circadian‐linked features may reflect an underlying disturbance of diurnal rhythm. Variability in subjective mood has been shown to correlate with objective variability in diurnal physiology, with mood being particularly sensitive to shifts in the phase and depth of sleep [39]. Together, these findings suggest that mixed features may represent a convergence of atypical and melancholic symptoms, each associated with bipolarity.

The link between suicidal ideation and mixed features echoes robust literature documenting higher suicidality rates in mixed presentations [10, 35], likely reflecting the increased psychological distress characteristics of mixed states [40]. The association with slow or mixed thoughts–which might appear counterintuitive given the DSM‐5 criterion on racing thoughts–may reflect the subjective experience of patients with racing thoughts who perceive their cognition as confused or jumbled, highlighting that the phenomenology of thought disturbances in mixed states may be more complex and show potential limitations of current diagnostic criteria.

The clinical correlates associated with mixed features in our study underscore the increased severity and complexity of this presentation. Higher rates of mood stabilizer use among patients with MDD with mixed features are consistent with treatment guidelines recommending mood stabilizers as a first‐line treatment for mixed presentation [41, 42]. The association with lifetime comorbid anxiety disorders, particularly panic disorder and specific phobia, align with evidence that mixed features are linked to heightened anxiety burden [10, 43]. The relationships with premenstrual syndrome symptoms and negative interpersonal relationships are particularly noteworthy, as both have been well‐documented in bipolar depression [44, 45]. These associations reinforce the notion that mixed features in MDD share neurobiological and clinical characteristics with bipolar disorder [43, 46].

The dose–response relationship between the number of mixed‐features symptoms and clinical characteristics further underscore the greater severity of MDE in MDD with the mixed‐features specifier. The associations with both dysthymia and a longer duration of the longest MDE suggest that patients with mixed features during their worst MDE are likely to experience depressive symptoms more persistently. This may contribute to greater impairment in social functioning [9]. Interpersonal difficulties—fewer friendships and more negative social interactions—may reflect the impact of prolonged depressive symptoms on social functioning. These interpersonal impairments could create a vicious cycle where social isolation and relationship conflicts exacerbate mood symptoms, highlighting the importance of addressing social functioning in patients with mixed features. Additionally, the association with generalized anxiety disorder is aligned with previous findings showing higher rates of co‐occurring anxiety disorders in mixed features [40]. Together, these findings emphasize the need to identify mixed features in MDD, as they signal a more complex and severe clinical profile that requires tailored treatment planning [10, 47].

This study has several limitations. First, the cross‐sectional design precludes causal inferences regarding the relationships between mixed features and clinical correlates. Second, because mixed features were ascertained only for the single, self‐reported worst MDE, the worst‐MDE‐based prevalence reported here is likely to underestimate the true lifetime occurrence of mixed features and may have diluted associations with clinical characteristics across the illness course. Third, retrospective self‐report of the worst lifetime MDE is subject to recall bias and subjective misinterpretation; this is particularly relevant for subjective symptoms, such as diurnal mood variation and slowed thinking, and is compounded by the complex phenomenology of mixed presentations. Fourth, because participants were drawn from a genome‐wide association study (KOMOGEN‐D) specifically designed to minimize genetic heterogeneity [48], eligibility was restricted to Korean women with four Korean grandparents and at least two MDEs. Although the design enhanced genetic and phenotypic homogeneity, it inherently constrains the generalizability of our findings to men, to non‐Korean populations, and to patients with a single MDE. Future studies are therefore needed to verify whether the present findings generalize to samples with characteristics different from those of the present cohort. Future longitudinal studies are needed to determine whether mixed features are present across all episodes experienced by a given patient and to prospectively verify their clinical consequences. Validation against medical records would further strengthen such evidence. In addition, although participants were recruited from 47 hospitals, our models did not account for potential center‐level clustering (e.g., site differences in diagnostic or treatment practices); given the low proportion of mixed features (~5%), the number of positive cases per site was too small to support stable cluster‐adjusted models, and the present estimates should therefore be interpreted without adjustment for differences across recruitment sites. Despite these limitations, this study provides the first large‐scale examination of mixed feature prevalence in an East Asian population and identifies specific within‐episode symptoms associated with mixed features during the worst MDE. Clinically, these findings underscore the importance of screening for mixed features in patients with recurrent MDD, particularly when they report increased weight, late insomnia, slow or mixed thoughts, suicidal ideation, or diurnal mood variation (worse mood in the morning). The associations between mixed features during the worst MDE and adverse outcomes highlight the prognostic significance of early identification and tailored treatment strategies—potentially including mood stabilizers rather than antidepressant monotherapy—to optimize outcomes.

## 5. Conclusions

In conclusion, this study provides important insights into the prevalence and clinical correlates of mixed features in Korean women with recurrent MDD. The 4.6% prevalence during the worst MDE aligns with other East Asian studies but is notably lower than that in Western populations. Five specific depressive symptoms emerged as unique markers of mixed features: increased weight, late insomnia, slow or mixed thoughts, suicidal ideation, and diurnal mood variation (worse in the morning). Mixed features were associated with greater mood stabilizer use, comorbid anxiety disorders, premenstrual syndrome symptoms, increased suicidality, and interpersonal dysfunction. These findings support the mixed‐features specifier as an indicator of bipolar vulnerability and worse clinical outcomes. Future cross‐cultural studies using culturally sensitive assessment tools are needed to clarify whether the observed East–West differences in mixed‐features prevalence reflect true phenomenological variations or methodological artifacts.

## Author Contributions

**C. Hyung Keun Park:** conceptualization, investigation, methodology, supervision, writing – original draft, writing – review and editing. **Chanhee Park:** conceptualization, formal analysis, methodology, project administration, writing – original draft, writing – review and editing. **Sang Jin Rhee and Sooyeon Min**: data curation, investigation, writing – review and editing. **Yoojin Song, Heon-Jeong Lee, Seunghee Won, Dongyun Lee, Do Hoon Kim, Ji Hyun Baek, Young-Hoon Ko, Kwang-Yeon Choi, Sung Joon Cho, Won Sub Kang, Young Min Choe, Sungwon Roh, Chun Il Park, Seoyoung Yoon, Hyung-Jun Yoon, Joonho Choi, Sangho Shin, Su Jeong Seong, and Hyeon Ah Lee**: investigation, writing – review and editing. **Jonathan Flint and Kenneth S. Kendler**: funding acquisition, methodology, supervision, writing – review and editing. **Yong Min Ahn:** conceptualization, investigation, supervision, writing – review and editing.

## Funding

The project was funded by the U.S. National Institutes of Health (Grant NIHU01MH126798) and the Wellcome Trust (Grant WT 200176/A/15/Z).

## Ethics Statement

The study protocol was approved by the Institutional Review Board at the University of California, Los Angeles; the ethics committees of all participating hospitals in Korea; and the Institutional Review Board of Asan Medical Center (2021‐1123). All procedures involving human participants were conducted in accordance with the Declaration of Helsinki (1964). All participants provided written informed consent after a full explanation of the study.

## Conflicts of Interest

The authors declare no conflicts of interest.

## Supporting Information

Additional supporting information can be found online in the Supporting Information section.

## Supporting information


**Supporting Information** The following supporting information is available for this article: Table S1: Interview questions used to assess the seven hypomanic symptoms of the DSM‐5 mixed features. Table S2: Interview questions used to assess mood‐related symptoms during the worst major depressive episode. Table S3: Univariate logistic regression testing the association between the mood‐related symptoms and the presence of mixed features in the worst major depressive episode. Table S4: Variance inflation factor for each variable entered into multivariable generalized linear model. Table S5: Effects of generalized linear models testing for association between the number of manic/hypomanic symptoms and lifetime clinical factors after controlling for the current mood state. Table S6: Effects of generalized linear models testing for association between the number of mixed features and lifetime clinical factors. Material S1: Associations between the number of manic/hypomanic symptoms and various clinical characteristics. Material S2: IRB approval numbers for each of the participating hospitals.

## Data Availability

The data that support the findings of this study are available from the corresponding author upon reasonable request.
