# Supplementary material for: Mixed Features in Recurrent Major Depressive Disorder: Prevalence, Risk Factors, and Clinical Consequences
Source: Depress Anxiety. 2026 Aug 30;2026:7452200. doi: 10.1155/da/7452200 (PMC13526251; doi:10.1155/da/7452200)
Supplement: Supplementary file 1 [file DA-2026-7452200-s001.docx]

**Supplementary Table 1**. Interview questions used to assess the seven (hypo)manic symptoms of the DSM-5 mixed features.

| Manic/hypomanic symptoms | Questions |
| --- | --- |
| Elevated, expansive mood | Did you feel elevated, expansive mood? |
| Inflated self-esteem or grandiosity | Did you feel inflated self-esteem or grandiosity? |
| More talkative than usual or pressure to keep talking | Did you talk a lot more than is normal for you? When talking with you, did other people have trouble getting a word in edgewise? |
| Flight of ideas or subjective experience that thoughts are racing | Were your thoughts often racing through your head? |
| Increase in energy or goal-directed activity (either socially at work or school, or sexually) | Did you have increased energy or goal directed activity (either socially at work or school, or sexually)? |
| Increased or excessive involvement in activities that have a high potential for painful consequences | Did you have increased or excessive involvement in activities that have a high potential for painful consequences? (e.g., engaging in unrestrained buying sprees, sexual indiscretions, foolish business investments) |
| Decreased need for sleep | Did you feel decreased need for sleep? (feeling rested despite sleeping less than usual; to be contrasted with insomnia) |

**Supplementary Table 2**. Interview questions used to assess mood-related symptoms during the worst major depressive episode.

| Mood-related symptoms in the worst MDE | Questions |
| --- | --- |
| Hopelessness | Did you feel hopeless about things? |
| Crying | Did you cry a lot? |
| Pathological sadness | Were your feelings of (sadness, depression, blueness) different from the usual feelings you would get after someone close to you died or from a sad movie or sad story? |
| Diminished or loss of interest | Were there still some things you could enjoy or be interested in, or did you lose your interest and enjoyment in nearly all activities? |
| Lack of reactivity to pleasurable stimuli | Did you lose the ability to enjoy having good things happen to you, like winning something or being praised or complimented? |
| Decreased libido | Was your interest in sex a lot less than usual? |
| Decreased weight | Did you lose weight without trying to, as much as one kilogram a week for at least two weeks? |
| Increased weight | Did your eating increase so much that you gained weight as much as one kilo a week for at least two weeks? |
| Decreased appetite | Did you have less appetite than usual almost every day for at least two weeks? |
| Increased appetite | Did you have a much larger appetite than is usual for you almost every day for two weeks or more? |
| Insomnia | Did you have trouble sleeping almost every night for two weeks or more - either trouble falling asleep, waking in the middle of the night, or waking up too early? |
| Late insomnia | Did you often wake up in the early morning before you wanted to get up and be unable to get back to sleep? |
| Hypersomnia | Were you sleeping too much almost every day? |
| Psychomotor agitation | Did you have to be moving all the time - that is, you couldn't sit still and paced up and down or couldn't keep your hands still when sitting? |
| Nervousness | Did you often feel nervous, jittery or anxious? |
| Psychomotor retardation | Did you talk or move more slowly than is normal for you almost every day for at least two weeks? |
| Slow or mixed thoughts | Did your thoughts come much slower than usual or seem mixed up? |
| Lack of energy or fatigue | During that time (when things were at their worst), did you lack energy or feel tired all the time nearly every day, even when you had not been working very hard? |
| Helplessness | Did you feel helpless much of the time? |
| Worthlessness | Did you feel worthless nearly every day for two weeks or longer? |
| Low self-worth | Did you feel that you were not as good as other people? |
| Low confidence | Did you have so little self-confidence that you wouldn't try to have your say about anything? |
| Feeling like failure | Did you often feel like you were a failure or a disgrace to your family? |
| Guilt | Did you feel guilty or remorseful? |
| Diminished concentration | Did you have a lot more trouble concentrating than is normal for you for two weeks or longer? |
| Indecisiveness | Were you unable to make up your mind about things you ordinarily had no trouble deciding about? |
| Impaired attention | Were you unable to read things that usually interest you or watch television or movies you usually liked, because you couldn't pay attention to them? |
| Death thought | Did you think a lot about death? |
| Suicidal ideation | Did you feel so low you thought a lot about committing suicide? |
| Suicide plan | Did you make a plan as to how you might do it? |
| Suicide attempt | Did you attempt suicide? |
| No initiative | Did you often feel like you could not get going and take the initiative to get things done? |
| Talking less | Did you talk a lot less than is normal to you? |
| Derealization | Did the world feel different to you, like everything had changed? |
| Diurnal variation | Most days did you feel particularly bad when you first got up, but felt better later in the day? |

**Supplementary Table 3.** Univariate logistic regression testing the association between the mood-related symptoms and the presence of mixed features in the worst major depressive episode

| Mood-related symptoms in the worst MDE | Mixed features | | Univariate logistic regression | | |
| --- | --- | --- | --- | --- | --- |
|  | Present  (*N* = 136) | Absent  (*N* = 2,796) | OR [95% CI] | *p* | *q* |
| Hopelessness | 126 | 2,475 | 1.56 [0.87, 3.26] | .46 | .58 |
| Crying | 117 | 2,305 | 1.30 [0.81, 2.19] | .37 | .44 |
| Pathological sadness | 125 | 2,528 | 1.04 [0.55, 2.23] | .92 | .93 |
| Diminished or loss of interest | 71 | 1,469 | 0.99 [0.70, 1.40] | .92 | .94 |
| Lack of reactivity to pleasurable stimuli | 108 | 2,388 | 0.62 [0.41, 0.97] | .01 | .07 |
| Decreased libido | 102 | 2,163 | 0.85 [0.57, 1.28] | .49 | .56 |
| Decreased weight | 76 | 1,436 | 1.16 [0.82, 1.65] | .34 | .55 |
| **Increased weight** | **47** | **433** | **2.92 [2.00, 4.20]** | **<.001** | **<.001** |
| Decreased appetite | 107 | 2,164 | 1.12 [0.74, 1.75] | .52 | .71 |
| **Increased appetite** | **45** | **474** | **2.41 [1.65, 3.47]** | **<.001** | **<.001** |
| Insomnia | 129 | 2,462 | 2.46 [1.23, 5.84] | .03 | .05 |
| **Late insomnia** | **121** | **2,171** | **2.27 [1.36, 4.07]** | **.01** | **.01** |
| Hypersomnia | 52 | 828 | 1.47 [1.02, 2.09] | .04 | .08 |
| **Psychomotor agitation** | **45** | **642** | **1.68 [1.15, 2.42]** | **<.001** | **.02** |
| **Nervousness** | **133** | **2,543** | **4.32 [1.62, 17.62]** | **.01** | **.03** |
| Psychomotor retardation | 59 | 1,032 | 1.34 [0.94, 1.90] | .08 | .19 |
| **Slow or mixed thoughts** | **115** | **1,918** | **2.70 [1.69, 4.54]** | **<.001** | **<.001** |
| Lack of energy or fatigue | 124 | 2,592 | 0.79 [0.45, 1.54] | .47 | .59 |
| Helplessness | 130 | 2,700 | 0.74 [0.34, 1.92] | .44 | .61 |
| Worthlessness | 122 | 2,374 | 1.50 [0.88, 2.75] | .22 | .27 |
| **Low self-worth** | **105** | **1,836** | **1.75 [1.18, 2.68]** | **.01** | **.02** |
| Low confidence | 115 | 2,227 | 1.37 [0.87, 2.26] | .22 | .31 |
| **Feeling like failure** | **109** | **1,971** | **1.74 [1.14, 2.74]** | **.02** | **.03** |
| **Guilt** | **122** | **2,273** | **1.98 [1.17, 3.63]** | **.02** | **.04** |
| Diminished concentration | 117 | 2,438 | 0.88 [0.55, 1.49] | .63 | .71 |
| Indecisiveness | 112 | 2,225 | 1.14 [0.74, 1.83] | .55 | .69 |
| Impaired attention | 102 | 2,115 | 0.95 [0.65, 1.43] | .80 | .87 |
| **Death thought** | **126** | **2,321** | **2.57 [1.41, 5.26]** | **.006** | **.02** |
| **Suicidal ideation** | **111** | **1,813** | **2.50 [1.63, 4.00]** | **<.001** | **.001** |
| **Suicide plan** | **77** | **1,122** | **1.94 [1.37, 2.76]** | **<.001** | **.001** |
| **Suicide attempt** | **54** | **733** | **1.85 [1.30, 2.63]** | **.001** | **.003** |
| No initiative | 116 | 2,528 | 0.61 [0.38, 1.02] | .04 | .08 |
| Talking less | 116 | 2,463 | 0.80 [0.50, 1.36] | .37 | .55 |
| **Derealization** | **97** | **1,509** | **2.09 [1.44, 3.09]** | **<.001** | **.001** |
| **Diurnal variation** | **66** | **1,006** | **1.65 [1.17, 2.33]** | **.003** | **.01** |

Note. Age was included as covariate. MDE = Major Depressive Episode; OR = Odds Ratio. CI = Confidence Interval. The false discovery rate (q-value) is reported for each p-value. A significance threshold of *q* < .05 was used. Totals may vary due to missing values in each variable. Significant results are in bold.

**Supplementary Table 4.** Variance inflation factor for each variable entered into multivariable generalized linear model.

| Variable | VIF |
| --- | --- |
|  |  |
| Increased weight | 2.10 |
| Increased appetite | 2.15 |
| Late insomnia | 1.01 |
| Agitation | 1.03 |
| Nervousness | 1.02 |
| Slow or mixed thoughts | 1.05 |
| Low self-worth | 1.25 |
| Feeling like failure | 1.26 |
| Guilt | 1.17 |
| Death thought | 1.50 |
| Suicidal ideation | 1.72 |
| Suicide plan | 1.43 |
| Suicide attempt | 1.27 |
| Derealization | 1.08 |
| Diurnal variation | 1.03 |

Note. Age was included as a covariate. OR = Odds Ratio, CI = Confidence Interval, LL = Lower Limit, UL = Upper Limit. Significant results are in bold.

**Supplementary Table 5.** Effects of generalized linear models testing for association between the mixed features and lifetime clinical factors after controlling for the current mood state.

| Variable | Estimate | 95% CI | | *p* | *q* |
| --- | --- | --- | --- | --- | --- |
|  |  | LLCI | ULCI |  |  |
| Lifetime use of psychotropics^a^ |  |  |  |  |  |
| Antipsychotics | 0.86 | 0.27 | 2.76 | .80 | .88 |
| **Mood stabilizers** | **2.33** | **1.49** | **3.64** | **<.001** | **.001** |
| Antidepressants | 0.90 | 0.36 | 2.25 | .82 | .88 |
| Other psychotropics | 0.77 | 0.54 | 1.09 | .14 | .26 |
| Current use of psychotropics |  |  |  |  |  |
| Antipsychotics | 0.86 | 0.27 | 2.76 | .80 | .88 |
| **Mood stabilizers** | **2.68** | **1.71** | **4.20** | **<.001** | **<.001** |
| Antidepressants | 1.06 | 0.55 | 2.06 | .86 | .89 |
| Other psychotropics | 0.83 | 0.58 | 1.17 | .29 | .41 |
| Psychiatric comorbidity |  |  |  |  |  |
| Dysthymia^a^ | 1.41 | 0.96 | 2.05 | .08 | .18 |
| **Premenstrual syndrome symptoms^b^** | **1.28** | **1.13** | **1.44** | **<.001** | **.001** |
| Generalized anxiety disorder^a^ | 1.52 | 1.07 | 2.15 | .02 | .05 |
| **Panic^a^** | **2.20** | **1.54** | **3.14** | **<.001** | **<.001** |
| **Phobia^a^** | **2.56** | **1.46** | **4.48** | **.001** | **.004** |
| Clinical factors |  |  |  |  |  |
| Number of depressive episodes^b^ | 0.95 | 0.83 | 1.08 | .43 | .58 |
| Duration of the longest depressive episode^b^ | 1.12 | 0.92 | 1.37 | .24 | .38 |
| Hospitalization^a^ | 1.26 | 0.87 | 1.83 | .23 | .38 |
| **Neuroticism^b^** | **1.14** | **1.08** | **1.21** | **<.001** | **<.001** |
| Suicidality |  |  |  |  |  |
| Number of suicide attempts^b^ | 0.99 | 0.79 | 1.23 | .90 | .90 |
| **Damage by suicide attempts^b^** | **1.63** | **1.24** | **2.15** | **<.001** | **.002** |
| Social life  Friends |  |  |  |  |  |
| Frequency of seeing friends^c^ | -0.18 | -0.49 | 0.14 | .28 | .41 |
| Number of friends^b^ | 0.89 | 0.78 | 1.02 | .11 | .21 |
| Positive interaction^c^ | 0.05 | -0.14 | 0.24 | .62 | .81 |
| Negative interaction^c^ | 0.31 | -0.02 | 0.63 | .07 | .16 |
| Relatives |  |  |  |  |  |
| Contact with relatives^c^ | -0.04 | -0.36 | 0.28 | .81 | .88 |
| Positive interaction^c^ | -0.20 | -0.47 | 0.08 | .16 | .29 |
| Negative interaction^c^ | 0.70 | 0.27 | 1.13 | **.001** | **.005** |
| Spouse |  |  |  |  |  |
| Positive interaction^c^ | -1.74 | -3.01 | -0.48 | **.01** | **.02** |
| Negative interaction^c^ | 1.81 | 0.74 | 2.88 | **.001** | **.004** |
| Social Club^c^ | 0.07 | -0.24 | 0.38 | .66 | .83 |

Note. Age and current mood state were included as a covariate. CI = Confidence Interval. The false discovery rate (q-value) is reported for each p-value. A significance threshold of *q* < .05 was used. Significant results are in bold. Estimates were reported for the three types of regression models: linear regression (standardized parameter estimates, β); logistic regression (OR); quasi-Poisson regression (RR). ORs and RRs were calculated by exponentiating the regression coefficients.

^a^Logistic regression was used.

^b^Quasi-poisson regression was used.

^c^Linear regression was used.

**Supplementary Materials 1**. **Associations between the number of manic/hypomanic symptoms and various clinical characteristics.**

The associations between the number of mixed features symptoms and a range of clinical characteristics are shown in Supplementary Table 6. Overall, most results were largely consistent with those examining the association between the presence of mixed features (binary) and clinical characteristics; however, additional significant associations emerged for variables that did not show significant relationships with the binary presence of mixed features.

A greater number of manic/hypomanic symptoms constituting the mixed features was associated with a higher likelihood of dysthymia (OR = 1.21, 95% CI = 1.10–1.32, *q* < .001) and general anxiety disorder (OR = 1.30, 95% CI = 1.19–1.41, *q* < .001). Furthermore, a greater number of manic/hypomanic symptoms were associated with longer duration of the longest major depressive episode (RR = 1.08, 95% CI = 1.03–1.13, *q* = .002).

Finally, patients who experienced more manic/hypomanic symptoms during their worst MDE tended to have fewer friends (RR = 0.96, 95% CI = 0.93–0.99, *q* = .01) and more negative interactions with friends (β = 0.12, 95% CI = 0.04–1.19, *q* = .005).

**Supplementary Table 6.** Effects of generalized linear models testing for association between the number of mixed features symptoms and lifetime clinical factors.

| Variable | Estimate | 95% CI | | *p* | *q* |
| --- | --- | --- | --- | --- | --- |
|  |  | LLCI | ULCI |  |  |
| Lifetime use of psychotropics^a^ |  |  |  |  |  |
| Antipsychotics | 1.04 | 0.81 | 1.31 | .73 | .80 |
| **Mood stabilizers** | **1.16** | **1.02** | **1.30** | **.02** | **.03** |
| Antidepressants | 1.07 | 0.87 | 1.36 | .55 | .63 |
| Other psychotropics | 0.96 | 0.89 | 1.04 | .29 | .44 |
| Current use of psychotropics |  |  |  |  |  |
| Antipsychotics | 1.01 | 0.78 | 1.27 | .93 | .95 |
| **Mood stabilizers** | **1.27** | **1.12** | **1.43** | **<.001** | **<.001** |
| Antidepressants | 1.16 | 0.99 | 1.36 | .07 | .13 |
| Other psychotropics | 0.97 | 0.90 | 1.05 | .49 | .59 |
| Psychiatric comorbidity |  |  |  |  |  |
| **Dysthymia^a^** | **1.21** | **1.10** | **1.32** | **<.001** | **<.001** |
| **Premenstrual syndrome symptoms^b^** | **1.08** | **1.04** | **1.11** | **<.001** | **<.001** |
| **Generalized anxiety disorder^a^** | **1.30** | **1.19** | **1.41** | **<.001** | **<.001** |
| **Panic^a^** | **1.24** | **1.13** | **1.35** | **<.001** | **<.001** |
| **Phobia^a^** | **1.35** | **1.21** | **1.51** | **<.001** | **<.001** |
| Clinical factors |  |  |  |  |  |
| Number of depressive episodes^b^ | 1.01 | 0.98 | 1.04 | .36 | .50 |
| **Duration of the longest depressive episode^b^** | **1.08** | **1.03** | **1.13** | **.001** | **.002** |
| **Current mood state^c^** | **2.36** | **1.39** | **3.04** | **.007** | **.02** |
| Hospitalization^a^ | 0.99 | 0.98 | 1.01 | .26 | .41 |
| **Neuroticism^b^** | **1.07** | **1.05** | **1.08** | **<.001** | **<.001** |
| Suicidality |  |  |  |  |  |
| Number of suicide attempts^b^ | 1.02 | 0.98 | 1.06 | .95 | .95 |
| **Damage by suicide attempts^b^** | **1.18** | **1.10** | **1.26** | **<.001** | **<.001** |
| Social life  Friends |  |  |  |  |  |
| Frequency of seeing friends^c^ | −0.20 | −0.08 | −0.01 | .08 | .13 |
| **Number of friends^b^** | **0.96** | **0.93** | **0.99** | **.004** | **.01** |
| Positive interaction^c^ | 0.04 | −0.02 | 0.06 | .36 | .50 |
| **Negative interaction^c^** | **0.12** | **0.04** | **0.19** | **.002** | **.005** |
| Relatives |  |  |  |  |  |
| Contact with relatives^c^ | −0.03 | −0.10 | 0.05 | .49 | .59 |
| Positive interaction^c^ | −0.01 | −0.07 | 0.05 | .74 | .80 |
| **Negative interaction^c^** | **0.20** | **0.10** | **0.29** | **<.001** | **<.001** |
| Spouse |  |  |  |  |  |
| **Positive interaction^c^** | **−0.59** | **−0.89** | **−0.31** | **<.001** | **<.001** |
| **Negative interaction^c^** | **0.33** | **0.09** | **0.57** | **.007** | **.02** |
| Social Club^c^ | −0.03 | −0.10 | 0.04 | .46 | .59 |

Note. Age was included as covariate. CI = Confidence Interval. The false discovery rate (q-value) is reported for each p-value. A q-value greater than .05 was set as the statistical significance threshold. Significant results are in bold. Estimates are reported for the three types of regression models: linear regression (standardized parameter estimates, β); logistic regression (OR); quasi-Poisson regression (RR). ORs and RRs were calculated by exponentiating the regression coefficients.

^a^Logistic regression was used.

^b^Quasi-poisson regression was used.

^c^Linear regression was used.

**Supplementary Materials 2. IRB approval numbers for each of the participating hospitals.**

CHA Bundang Medical Center: 2021-08-062

CHA Ilsan Medical Center: 2025-01-009

Chosun University Hospital: 2022-01-002-015

Chungnam National University Hospital: CNUH 2022-09-066

Chungnam National University Sejong Hospital: CNUSH 2023-03-010-004

Daegu Catholic University Medical Center: DCUMC 2021-06-022-016

Daejeon Eulji University Hospital: EMC 2024-07-007

Dankook University Hospital: DKUH 2021-07-009

Ewha Womans University Mokdong Hospital: 2021-07-012-001

Gachon University Gil Medical Center: GCIRB2021-310

Gyeongsang National University Changwon Hospital: GNUCH 2021-07-020

Hallym University Chuncheon Sacred Heart Hospital: 2023-08-007

Hallym University Dongtan Sacred Heart Hospital: 2022-11-018

Hanyang University Guri Hospital: 2021-07-045

Hanyang University Seoul Hospital: 2021-08-012

Inha University Hospital: 2021-06-030

Kangbuk Samsung Hospital: 2021-11-013

Kangdong Sacred Heart Hospital: IRB-2021-07-016

Kangwon National University Hospital: KNUH 2023-03-014

Konyang University Hospital: KYUH 2023-08-009-027

Korea University Anam Hospital: 2021AN0358

Korea University Ansan Hospital: 2021AS0273

Korea University Guro Hospital: 2021GR0397

Kosin University Gospel Hospital: KUGH202205048

Kyung Hee University Hospital: 2021-11-070

Kyungpook National University Hospital: 2021-08-007

Myongji Hospital: MJH 2021-08-028

National Health Insurance Service Ilsan Hospital: 2021-06-029

National Medical Center: NMC 2022-08-093-010

Nowon Eulji University Hospital: 2021-06-006

Presbyterian Medical Center: 2024-04-011

Pusan National University Hospital: 2207-001-116

Pusan National University Yangsan Hospital: 05-2121-244

Samsung Medical Center: 2021-06-211

Seoul Medical Center: 2022-01-005

Seoul Metropolitan Government-Seoul National University Boramae Medical Center: 30-2022-82

Seoul National University Bundang Hospital: B-2108-702-403

Severance Hospital: 4-2021-0728

Soonchunhyang University Cheonan Hospital: 2021-07-051

Uijeongbu Eulji University Hospital: UEMC 2021-07-014

Wonju Severance Christian Hospital: CR321069

Wonkwang University Hospital: WKUH 2024-10-016

Wonkwang University Sanbon H`ospital: WMCSB 202508-11

Yeungnam University Medical Center: YUMC IRB 2023-11-048

Yong-In Mental Hospital: 2024-14
